# Long-time sickness absence among parents of pre-school children with cerebral palsy, spina bifida and down syndrome: a longitudinal study

**DOI:** 10.1186/s12887-016-0774-8

**Published:** 2017-01-18

**Authors:** Idunn Brekke, Elena Albertini Früh, Lisbeth Gravdal Kvarme, Henrik Holmstrøm

**Affiliations:** 1 Faculty of Health Sciences – Department of Nursing and Health Promotion, Oslo and Akershus University College of Applied Sciences, Post Box 4, St. Olavs plass, N-0130 Oslo, Norway; 2 Department of Paediatrics, Oslo University Hospital, Oslo, Norway

**Keywords:** Children with special needs, Down syndrome, Cerebral palsy, Spina bifida, Parental health, Sickness absence

## Abstract

**Background:**

Taking care of a child with special needs can be draining and difficult and require a lot of parental time and resources. The present study investigated the long-term sickness absence of parents who have children with spina bifida, cerebral palsy and Down syndrome compared to that of parents without a child with special needs.

**Methods:**

The sample consisted of primiparae women who gave birth between 2001 and 2005 and the fathers of the children (*N* = 202,593). Data were obtained from the Medical Birth Registry of Norway (MBRN), which is linked to the Central Population Register, education and income registries and Historical Event Database (FD-Trygd) of Statistics Norway (SSB). The linkage data provide longitudinal data, together with annual updates on children and their parents. Statistical analyses were performed using difference-in-difference (DD) study design.

**Results:**

Caring for a child with special needs affected maternal sickness absence, particularly in the first year after the birth. The level of sickness absence of mothers caring for a child with spina bifida and cerebral palsy was greater than that of mothers caring for a child with Down syndrome. In contrast, the sickness absence of fathers caring for a child with special needs was, on average, comparable to that of fathers without a special-needs child in the post-birth period.

**Conclusions:**

Caring for a child with special needs affected the long-term sickness absence of mothers but not fathers. The findings indicate that the burden of care in the case of children with special needs falls especially on the mother.

## Background

Chronically ill children and children with disabilities require a great deal of parental investment because their special needs [1] (e.g. specialised medical care and high numbers of medical visits), which are frequently greater than those of children without special needs. These additional needs can be stressful and burdensome for parents [1–3]. Barriers, such as a lack of coordination among service agencies [4] and poor and inaccurate information regarding available services, can be overwhelming obstacles for parents [5]. The intense nature of the care and responsibilities can have an adverse effect on the parents’ health and well-being, particularly that of the mothers [6–8]. Although the literature on the sickness absence of parents with a special-needs child is scarce, one study reported that, in general, it was higher than that of parents without a special-needs child and that the sickness absence of mothers of a special needs child was higher than that of the fathers [6]. Mothers of children with mild and moderate/severe care needs were reported to be more likely to have long-term sick leave compared with mothers in general, particularly due to mental health problems [9]. On the other hand, a Swedish study showed that the number of sickness benefit days of parents of children with Down syndrome did not differ from those of other parents [10].

The aforementioned studies included diverse special needs groups, potentially obscuring differences between children with diverse needs. As shown earlier, the challenges parents face and how they handle these may differ according to the nature of the child’s disability [3, 11, 12]. The type of special needs, including the level of care the child requires, may give rise to distinct parental challenges and have different effects on parental health and sickness absence. Previous research showed that the parents of children with Down syndrome were often less stressed and had better health than the parents of children with other special needs [11, 13]. Other studies demonstrated that children with cerebral palsy usually required a high degree of parental care [14], and that the level of caregiving was strongly associated with increased parental stress [15] and impaired psychological and physical health, particularly among mothers [16]. Parenting a child with spina bifida also required specialty care and involved a high level of parental attention, resulting in psychological suffering among the mothers of the children [17–19].

Previous research reported that the psychological well-being profiles of parents who had children with Down syndrome were typically better than those of parents caring for children with other types of disabilities [11, 13]. We hypothesised that parents of children with Downs syndrome would be less negatively affected in terms of long-term sick leave compared to parents of children with cerebral palsy and spina bifida. Furthermore, we expected that the child’s special care needs would fall primarily on the mother. Mothers are often the primary caretaker in the family [20, 21], and research indicates that caring for a child with special needs exerts greater psychological effects on mothers than on fathers [6–8]. The present study included the parents of children with cerebral palsy, spina bifida and Down syndrome and the parents of children without special care needs. Based on national register data, the study aimed to investigate whether having a child with special needs affected long-term sickness absence over time compared to the general population of parents. Secondary purposes of the study were to analyse potential differences between the long-term sickness absence of the parents of children with spina bifida/cerebral palsy and those with Down syndrome and between the mothers and fathers.

## Methods

### Study design and data sources

The sample in the present study consisted of primiparae women who gave birth between 2001 and 2005 and the fathers of the children (*N* = 202, 593). Data were obtained from the Medical Birth Registry of Norway (MBRN), which contains information on all births in Norway, and is linked to the Central Population Register, education and income registries and Historical Event Database of (FD-Trygd) of Statistics Norway (SSB). The linkage data provide longitudinal data, together with annual updates on children and their parents. The FD-Trygd panel database contains information on the country of origin, age, gender, labour market outcomes and welfare benefits for all individuals in Norway. The MBRN provides information on children with birth defects and serious illnesses. Diagnoses, such as spina bifida and Down syndrome, are usually registered at birth, but cerebral palsy is detected after birth. Therefore, information on auxiliary benefits and associated diagnoses (ICD-10) derived from FD-Trygd was used to identify the parents of children with cerebral palsy. Auxiliary benefits are granted to individuals with a long-term illness and long-term care needs or personal nursing. These are granted based on the care needs of the recipient and are independent of the person’s income. [22]. To be included in the analyses, the parents had to be employed.

#### Outcome measures

Information on individual sick leave was obtained from the FD-Trygd SSB database. In the register, sick leave due to the parents’s own sickness is distinguished from an absence related to the sickness of the children. The dependent variable in the current study was the number of parents own sickness absence days (i.e. a duration measure of sickness absence was used). Long-term sickness absence was measured because the recorded data included sickness absence of 17 days or more.

#### Diagnosis

The parents were divided in three different groups: parents of children without special needs (i.e. having children born without a birth defect and children that did not receive auxiliary benefits), parents of children with cerebral palsy and spina bifida and parents of children with Down syndrome. The classification is based on ICD-10 diagnosis codes. Due to the low number of recorded spina bifida and cerebral palsy cases, both groups were merged into one group. The diagnoses of Down syndrome and spina bifida were derived from the MBRN, and the diagnosis of cerebral palsy registered after birth was derived from FD-trygd SSB database.

#### Controls

*Younger siblings* born in the observation period were employed as a dummy variable, taking the value 1 if there were younger siblings in the household and 0 otherwise*. Country of origin* was determined by three dummy variables (Norway, Western countries and non-Western countries). The *age* of the parents was measured as the number of years. *Marital status* (i.e. whether the mother and father were married) was also used as a dummy variable. *Place of residence* was used as a dummy variable, taking the value 0 if the parents lived in Oslo (the capital) and 1 otherwise. *Educational level* was divided into four levels: compulsory school or lower, upper secondary school, bachelor’s level and master’s level/PhD. Missing educational information was included as a separate category. A dummy variable was used for children who *died* within the observation period, taking the value 1 if the child died and 0 otherwise. The *unemployment rate* in the local labour market (county) was recorded for each year. *Birth cohorts year dummies* and *number of days employed* were also controlled for.

#### Statistical analysis

A difference-in-difference (DD) study design was used to analyse the impact of having a child with special needs. Long-term maternal and paternal sickness absence prior to and after the birth of a child with special needs was compared with that of matched control groups who had a child without special needs over the same period. The analyses of long-term sickness absence were performed using Poisson regression, which models the frequency of event counts or the event rate. Poisson regression is a special case of a generalised linear model with a log link, also called a log-linear model, and is often used for the analysis of rare events [23]. It assumes that the outcome variable follows a Poisson distribution [23]. As the dependent variables were the number of days absent, Poisson regression was appropriate. All the results are presented as marginal effects, evaluated at the mean of the explanatory variables. Statistical analysis was performed using Stata® 13. One important assumption when using a DD design is that pre-trends in the dependent variable used for treatments and controls are similar. Using the DD approach, pre-trends in parental long-term sickness absence among the test group (i.e. parents who subsequently had a child with special needs) and controls (i.e. parents who did not subsequently have a child with special needs) were investigated. The results showed that the sickness absence trend among parents caring for a child with special needs and parents caring for a child without special needs was very similar prior to birth (results not shown).

## Results

Table 1 lists the means and proportions across the different groups of parents, separately for mothers and fathers.

**Table 1:** Sociodemographic variables of the mothers and fathers of children with and without special needs

	Mothers			Fathers		
	Child without special needs	Child with cerebral palsy/spina bifida	Child with Down syndrome	Child without special needs	Child with cerebral palsy/spina bifida	Child with Down syndrome
Age, mean (SD), yrs	27.78 (4.89)	27.50 (4.80)	30.42 (6.07)	30.99 (5.96)	31.09 (6.21)	33.57 (6.54)
Immigrant background						
Native Norwegian (%)	80.5	90.1	80.2	80.5	79.5	88.5
Non-western (%)	10.8	7.4	9.8	9.3	9.6	6.5
Western (%)	10.4	2.4	9.8	10.2	10.8	4.9
Married (%)	54.3	50.6	50.5	54.9	49.4	49.1
Younger children in the household (%)	37.1	41.9	37.3	37.1	40.9	31.1
Educational level						
Compulsory school or less (%)	17.8	20.9	15.3	19.3	22.9	13.1
Upper secondary school (%)	34.7	40.7	32.9	44.9	51.8	60.6
Bachelor’s level (%)	36.8	30.8	43.9	23.4	16.9	16.3
Master’s level (%)	8.6	4.9	6.5	10.4	3.6	6.5
Unknown (%)	3.8	2.4	1.1	2.1	4.8	3.2
Lives in the capital (%)	19.4	6.1	25.2	18.8	3.6	16.3
Unemployment rate, mean (SD)	3.5 (0.84)	3.1 (0.78)	3.5 (1.0)	3.49 (0.84)	3.17 (0.83)	3.3 (0.95)
*n*=	101667	81	91	100582	81	91

The first aim of the study was to investigate whether having a child with special needs affected long-term sickness absence over time. Table 2 presents the regression results for long-term sickness absence for mothers and fathers separately as compared to those of the general population.

**Table 2:** Poisson regression analyses of the sickness absence of employed mother and fathers, with the number of sickness days as the dependent variable. The sample consisted of primiparae women and fathers, birth cohort (2001–2005)

*n* = (person-years)						
	Coeff	SE	*P* value	Coeff	SE	*P* value
	Mothers, *n* = 546784			Fathers, *n* = 608266		
Diagnosis children						
Cerebral palsy/spina bifida	16.95	7.76	0.029	7.91	4.68	0.091
Down syndrome	−2.95	5.20	0.570	8.80	7.08	0.214
Without special needs	reference			reference		
Time						
2 years prior to the birth	−29.53	0.42	0.000	−0.95	0.20	0.000
Birth year	20.09	0.21	0.000	0.56	0.17	0.001
1 year after the birth	−18.57	0.34	0.000	0.28	0.17	0.110
2 years after the birth	4.97	0.27	0.000	1.03	0.19	0.000
3 years after the birth	4.26	0.28	0.000	1.34	0.19	0.000
4 years after the birth	−0.85	0.31	0.006	1.99	0.20	0.000
1 year prior to the birth	reference			reference		
Interactions						
Cerebral palsy/spina bifida × 2 years prior to the birth	6.83	11.27	0.544	−2.64	5.40	0.624
Cerebral palsy/spina bifida × year of the birth	−1.19	5.91	0.840	5.87	3.40	0.084
Cerebral palsy/spina bifida × 1 year after the birth	**35.42**	7.12	0.000	4.72	3.66	0.197
Cerebral palsy/spina bifida × 2 years after the birth	9.45	7.35	0.199	1.81	4.77	0.703
Cerebral palsy/spina bifida × 3 years after the birth	3.52	7.40	0.634	0.63	4.18	0.880
Cerebral palsy/spina bifida × 4 years after the birth	6.23	7.60	0.412	4.88	3.70	0.187
Down syndrome × 2 years prior to the birth	−4.83	11.55	0.676	4.47	5.37	0.406
Down syndrome × year of the birth	12.65	6.43	0.049	3.94	4.37	0.367
Down syndrome × 1 year after the birth	**26.16**	7.79	0.001	−5.56	5.01	0.267
Down syndrome × 2 years after the birth	11.70	8.09	0.148	−11.74	8.24	0.154
Down syndrome × 3 years after the birth	9.36	7.83	0.232	−5.61	6.43	0.383
Down syndrome × 4 years after the birth	16.11	8.56	0.060	−7.02	8.48	0.408
Pseudu R2	0.083			0.064		

*Note*: Age, age squared, immigrant background, length of residency, marital status, younger children in the household, birth cohort, place of residency, number of days employed and the unemployment rate are included in the models

**Fig. 1:**
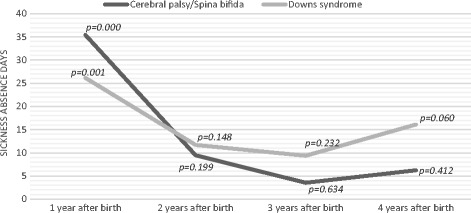
Difference in the sickness absence of mothers with and without a child with special needs (control group), based on estimates from Table 2. *Note: p < 0.05 indicates a significant difference in the sickness absence prior to and after the birth compared to that of the control group*

The model included several controls and an interaction term between the child’s diagnosis and the year, which was used as a dummy variable. The interaction term shows whether the differences between parents with a child with special needs and parents with a child without special needs in the year prior to birth, changed during the post-birth period. The results demonstrated that the long-term sickness absence of mothers caring for a child with special needs increased substantially in the post-birth period compared to that of mothers of a child without special needs, particularly during the first year after the birth. On average, the first year after the birth, mothers who gave birth to a child with cerebral palsy or spina bifida had 35 more sick leave days than mothers who had children without special needs (*p* = 0.000). Table 2 shows that mothers caring for a child with Down syndrome had, on average, 26 more sick days during the first year after the birth than mothers of children without special needs (*p* = 0.001). There were no significant differences between the test and control group 2 and 3 years after the birth, but there was a tendency towards increased sick leave among mothers 4 years after the birth of a child with Downs syndrome (*p* = 0.06). This result is also illustrated in Fig. 1. The results support our hypothesis that caring for a child with special needs has a negative impact on maternal sickness absence.

The secondary purposes of the study were to analyse potential differences between parents of children with spina bifida/cerebral palsy and Down syndrome. The results of *t*-tests^1^ of the potential effect of the type of special needs on maternal sickness absence demonstrated that 1 year after the birth, mothers of children with spina bifida and Cerebral palsy had 9 more sick days, on average, than mothers of children with Down syndrome. Therefore, the *t-*tests pointed to significant differences in the level of sickness absence between the two diagnosis the first year after birth (*t* > 1.96).

Finally, the study aimed to investigate differences in sickness absence between mothers and fathers. As shown in Table 2, the sickness absences among fathers caring for a child with spina bifida, cerebral palsy and Down syndrome did not increase significantly compared to that of fathers with a child without special needs in the post-birth period. The results indicate that caring for a child with special needs seems to affect mothers’ but not fathers’ sickness absence.

## Discussion

The results showed that caring for a child with special needs seems to affect the level of maternal sickness absence, particularly in the first year after the birth. We cannot be completely sure that the increased sickness absence is caused by the caring burden, however the results are in line with previous research showing that mothers caring for a child with special needs are at substantial risk of a long-term sick leave due to psychiatric disorders [9]. Moreover, a recent review article also indicates that caring for a child with special needs has adverse effects on mothers’ health [3]. Thus, there is reasonable to suggest that intensified care burden affect mother’s health, which in turn will increase long-term sickness absence. In addition, struggling with the health and social care system, and challenges in everyday life among these families [4, 5] might also result in maternal sickness absence due to distress and time demanding tasks related to having a child with special needs.

Furthermore, the results demonstrated that sickness absence was greater among mothers caring for a child with spina bifida and cerebral palsy than among mothers of children with Down syndrome. The sickness absence of the fathers caring for a child with cerebral palsy, spina bifida and Down syndrome was similar to that of the fathers of a child without special needs.

The aim of this article was to shed light on the health and sickness absence of parents caring for pre-school children with spina bifida, cerebral palsy and Down syndrome. An additional goal was to analyse differences in parental sickness absence according to the type of special needs the child had and differences between the levels of absenteeism of the mothers versus the fathers. The results showed that caring for a child with special needs affected maternal sick leave, with the mothers of pre-school children who had spina bifida, cerebral palsy and Down syndrome having higher sickness absence than the mothers of children with no special needs. This result is in line with that of previous research [6, 9]. It supports the notion that caring for a child with special needs impairs maternal health, resulting in an increased level of sick leave. The findings also illustrated that caring for a child with special needs affected only the mother’s level of sick leave, not that of the fathers, whose sickness absence levels were similar to those of the fathers of children without special needs. This finding is in accordance with that of previous studies [3, 6–8]. Mothers are often the primary caregiver [21, 24]. The results of the present study seem to indicate that the responsibility of caring for a child with special needs falls predominantly on the mothers.

The present study also showed that the sickness absence patterns among the parents varied according to the child’s age, with the greatest effects on maternal sick leave observed in the first year after the child was born. This applied to all the mothers, irrespective of the type of special needs of the child (i.e. spina bifida, cerebral palsy or Down syndrome). The first year may be the most critical, possibly due to the major adjustments that have to be made when the mothers return to work and the child starts in kindergarten.

The results also illustrated that the sickness absence patterns differed according to the type of special need, with the mothers of children with cerebral palsy and spina bifida having higher sickness absence levels than the mothers of children with Down syndrome. The results confirm previous research, which found that mothers of children with Down syndrome often fare better than mothers of children with other types of special needs [11, 13]. The higher levels of sickness absence among the mothers of children with cerebral palsy and spina bifida may be related to the fact that these children have physical disabilities. These may be particularly challenging when the child enters kindergarten. In the first year of life, many children with cerebral palsy have feeding difficulties due to oral motor dysfunction [25], in addition to other types of impairments, such as spasticity, dyskinesia and ataxia [26]. Children with spina bifida may have bladder and bowel dysfunction, which was found to be a stressor among parents [27]. Urinary tract infections were also reported to be common among children with spina bifida [28]. The increased level of stress and worry associated with the complex care needs of children with spina bifida or cerebral palsy may increase the level of maternal sick leave.

The strengths of this study are the large sample size and longitudinal design, with a wide range of sociodemographic, health and work-related pre- and post-birth data on both fathers and mothers who gave birth from 2001 to 2005. Cross-sectional studies have tended to dominate this area of research. The present study included both mothers and fathers, whereas previous research focused mainly on mothers. An additional strength of this study is the inclusion of data on children with different special needs. Thus, it was possible to analyse differences in parental sick leave according to the type of special needs the child had. The first limitation of this study is that the follow-up data included children only up to the age of 4 years. It is possible that the between-group differences in parental sick leave patterns observed herein may have changed when the child was older. For example, previous research indicated that stress among parents of children with Down syndrome increased over time [12, 29]. Second, the sample size of the spina bifida and cerebral palsy groups was rather small. Therefore, the two groups had to be merged into one group. A third limitation is that the data did not contain information on attendance allowances, which compensate for the loss of income related to care responsibilities^2^. Thus, the differences in sickness absence between the parents of a child with special needs and those of a child without special needs might be underestimated [2]. Finally, the last limitation is that in the first year after birth many women in Norway are on parental leave for large parts of this year. Thus, we are not able to compare sick leave across calendar years. However, the focus in the present article is differences in sick leave between parents with a child with special needs and parents with a healthy child. In this respect, the problems related to parental leave in year 1 is not problematic since the situation is comparable for the two groups of parents.

## Conclusions

The results of this study are broadly consistent with those of other studies, and they suggest that mothers of children with special needs take more sick leave than do mothers of children with no special needs. However, the fathers seem to be unaffected in terms of sickness absence. The study also shows that the first year after a child is born is a sensitive period, with the level of maternal sickness absence relatively high among mothers caring for a child with special needs, particularly among mothers of a child with spina bifida and cerebral palsy. In terms of specialised services and support programs, the findings of this study imply that the mothers of a child with special needs require support from the time the child is very young.

In terms of specialised services and support programmes, the findings of this study imply that the mothers of a child with special needs require support from the time the child is very young. The society should support caregivers and recognize their efforts in caring for their child so that the parents enjoy dignity and integrity [30]. The practionaire and specialised services must focus on aspects that promote health among these mothers, such as social support and guiding in access to resources and benefits. Early intervention by engaged professionals can make everyday life easier for these mothers, and are a necessity for their participation in the labor market [31], and will in turn most likely reduce mothers’ absence from work. Longitudinal studies of children with special needs are required to develop the knowledge base on parental health and coping as the child grows older. Future research in this field should pay more attention to parents caring for schoolchildren with special needs.

## Abbreviations

coeff: Coefficient
DD: Difference-in-difference
FD-Trygd: Historical event database
ICD-10: International statistical classification of diseases and related health problems
MBRN: Medical birth registry of Norway
*p* value: Probability value
SD: Standard deviation
SE: Standard error
SSB: Statistics Norway

## Footnotes

1
*T*-tests were conducted of the mothers of children with spina bifida/cerebral palsy and the mothers of children with Down syndrome (*t*-value = 1.96). T-values shown in bold indicate a significant difference in sickness absence.

2 In 2014, the mean attendance allowance received in Norway among parents caring for a child with special needs was 6506 EUR.
